# Community Outbreak of HIV Infection Linked to Injection Drug Use of Oxymorphone — Indiana, 2015

**Published:** 2015-05-01

**Authors:** Caitlin Conrad, Heather M. Bradley, Dita Broz, Swamy Buddha, Erika L. Chapman, Romeo R. Galang, Daniel Hillman, John Hon, Karen W. Hoover, Monita R. Patel, Andrea Perez, Philip J. Peters, Pam Pontones, Jeremy C. Roseberry, Michelle Sandoval, Jessica Shields, Jennifer Walthall, Dorothy Waterhouse, Paul J. Weidle, Hsiu Wu, Joan M. Duwve

**Affiliations:** 1 Indiana State Department of Health; 2 Division of HIV/AIDS Prevention, National Center for HIV/AIDS, Viral Hepatitis, STD, and TB Prevention, CDC; 3 Epidemic Intelligence Service, CDC; 4 Clark County Health Department, Jeffersonville, Indiana; 5 Indiana University Richard M. Fairbanks School of Public Health, Indianapolis, Indiana

On January 23, 2015, the Indiana State Department of Health (ISDH) began an ongoing investigation of an outbreak of human immunodeficiency virus (HIV) infection, after Indiana disease intervention specialists reported 11 confirmed HIV cases traced to a rural county in southeastern Indiana. Historically, fewer than five cases of HIV infection have been reported annually in this county. The majority of cases were in residents of the same community and were linked to syringe-sharing partners injecting the prescription opioid oxymorphone (a powerful oral semi-synthetic opioid analgesic). As of April 21, ISDH had diagnosed HIV infection in 135 persons (129 with confirmed HIV infection and six with preliminarily positive results from rapid HIV testing that were pending confirmatory testing) in a community of 4,200 persons (1).

The age range of the 135 patients is 18–57 years (mean = 35 years; median = 32 years); 74 (54.8%) are male. A small number of pregnant women were diagnosed with HIV infection and started on antiretroviral therapy during pregnancy. As of April 21, no infants had tested positive for HIV. Of the 135 persons with diagnosed HIV infection, 108 (80.0%) have reported injection drug use (IDU), four (3.0%) have reported no IDU, and 23 (17.0%) have not been interviewed to determine IDU status. Among the 108 who have reported IDU, all reported dissolving and injecting tablets of oxymorphone as their drug of choice. Some reported injecting other drugs, including methamphetamine and heroin. Ten (7.4%) female patients have been identified as commercial sex workers. Coinfection with hepatitis C virus has been diagnosed in 114 (84.4%) patients.

The patients were interviewed about syringe-sharing and sex partners, as well as any social contacts who also might have engaged in high risk behaviors. Those interviewed reported an average of nine syringe-sharing partners, sex partners, or other social contacts who might be at risk for HIV infection. Of the 373 contacts named as of April 21, a total of 247 (66.2%) had been located, 230 (61.7%) were tested, and 17 (4.6%) either declined testing or were not able to be tested. Of the 230 contacts who were tested, test results for 109 (47.4%) were HIV positive, and 121 (52.6%) were HIV negative. Of the 128 contacts who have not yet been located, 74 (57.8%) have been identified as syringe-sharing or sex partners, and 54 (42.2%) are social contacts regarded as at high risk for HIV infection.

Injection drug use in this community is a multi-generational activity, with as many as three generations of a family and multiple community members injecting together. IDU practices include crushing and cooking extended-release oxymorphone, most frequently 40 mg tablets not designed to resist crushing or dissolving. Syringes and drug preparation equipment are frequently shared (e.g., the drug is dissolved in nonsterile water and drawn up into an insulin syringe that is usually shared with others). The reported daily numbers of injections ranged from four to 15, with the reported number of injection partners ranging from one to six per injection event.

Like many other rural counties in the United States, the county has substantial unemployment (8.9%), a high proportion of adults who have not completed high school (21.3%), a substantial proportion of the population living in poverty (19%), and limited access to health care (1). This county consistently ranks among the lowest in the state for health indicators and life expectancy (2).

ISDH worked with the only health care provider in the immediate community, local health officials, law enforcement, community partners, regional health care providers and CDC to launch a comprehensive response to this outbreak. A public health emergency was declared on March 26 by executive order (3). The response has included a public education campaign, establishment of an incident command center and a community outreach center, short-term authorization of syringe exchange, and support for comprehensive medical care including HIV and hepatitis C virus care and treatment as well as substance abuse counseling and treatment. State and local health departments and academic partners, with the assistance of CDC, are working to implement and improve the community outreach programs supported by the executive order and to interrupt IDU-related HIV and hepatitis C virus transmission. Contact tracing by state and CDC disease intervention specialists continues to identify those potentially exposed.

This HIV outbreak involves a rural population, historically at low risk for HIV, in which HIV infection spread rapidly within a large network of persons who injected prescription opioids. The Indiana public health response includes implementing programs to contain the spread of HIV and hepatitis C virus, curb injection drug use, and concurrently build social resilience in the community. The outbreak highlights the vulnerability of many rural, resource-poor populations to drug use, misuse, and addiction, in the context of a high prevalence of unaddressed comorbid conditions (4). The outbreak also demonstrates the importance of timely HIV and Hepatitis C surveillance activities and rapid response to interrupt disease transmission. Finally, the outbreak points to the need for expanded mental health and substance use treatment programs in medically underserved rural areas (5).
